# Supplementary material for: Chiral derivatization-enabled discrimination and on-tissue detection of proteinogenic amino acids by ion mobility mass spectrometry
Source: Chem Sci. 2022 Nov 21;13(47):14114–23. doi: 10.1039/d2sc03604e (PMC9728562; doi:10.1039/d2sc03604e)
Supplement: SC-013-D2SC03604E-s001 [file SC-013-D2SC03604E-s001.pdf]

Electronic Supplementary Material (ESI) for Chemical Science.
This journal is © The Royal Society of Chemistry 2022

Electronic Supporting Information

Chiral derivatization-enabled discrimination and on-tissue detection of proteinogenic amino acids by ion mobility mass spectrometry

Chengyi Xie[a], Yanyan Chen[a], Xiaoxiao Wang[a], Yuanyuan Song[a], Yuting Shen[a], Xin Diao[a], Lin Zhu[a], Jianing Wang,[a,b,*] and Zongwei Cai,[a,*]

[a] State Key Laboratory of Environmental and Biological Analysis, Department of Chemistry, Hong Kong Baptist University, Hong Kong SAR, China

[b] Institute for Research and Continuing Education, Hong Kong Baptist University, Hong Kong SAR, China

Table of Contents

1.1 Animal preparation 3

1.2 Amino acids extraction from mouse brain tissue 3

1.3 Sample preparation for MALDI MSI 3

Table S1. Parameters of direct-infusion TIMS-MS in Tune 5

Table S2. Parameters of MALDI TIMS-MS in Tune 6

Table S3. Parameters in Tune for Q-TOF only mode 7

Table S4. Elution orders and *R* of FDAA-AA diastereomers obtained from TIMS-MS 8

Table S5. Lists of product ions from precursor ions $[FDAA\text{-}AA + 2Na - H]^+$. 9

Table S6. Extracting windows of *m/z* and mobility in Fig. S18 10

Fig. S1. Mass spectra of DL-Trp and DL-Ser (D: L = 1: 1) after FDAA derivatization 11

Fig. S2. Overlay EIMs of a single chiral amino acid standard after FDAA derivatization 12

Fig. S3. EIMs of Trp standards (100 μM) before derivatization. 13

Fig. S4. EIMs of 19 pairs of proteinogenic AA (D:L = 1:1) standards after FDVA derivatization. 14

Fig. S5. Mass spectra of AAs with multiple reaction sites (DL-Lys and DL-Tyr) 15

Fig. S6. EIMs of bis-derivatives of (A) Tyr and (B) Lys 16

Fig. S7. Determination of the reaction site of Tyr with FDAA by the combination of TIMS and tandem mass spectrometry (MS/MS) 17

Fig. S8. EIMs of mono-derivatives of Cys. 18

Fig. S9. Mass spectra of 38 mixed amino acids corresponding to Fig. 4. 19

Fig. S10. Separately listed EIMs of the mixture of 38 chiral AAs corresponding to Fig. 4 20

Fig. S11. EIMs of 15 AAs measured in the mouse brain extracts after FDAA derivatization 21

Fig. S12. An example of amino acid (Ala) identification in mouse brain extracts using FDAA derivatization by TIMS-MS. 22

Fig. S13. Mass spectra and EIMs of Tyr and Trp measured in the mouse brain extracts after FDAA derivatization 23

Fig. S14. The plot of peak area ratio against enantiomeric ratio of Lys after FDAA derivatization 24

Fig. S15. EIMs of *m/z* 418.040 ± 0.01 measured in the mouse brain section and Cys standards after FDAA derivatization 25

Fig. S16. Confirmation of FDAA-AAs observed in Fig. 6 by tandem mass spectrometry 26

Fig. S17. Mass spectra of DL-Trp and DL-Ser after FDAA derivatization acquired by MALDI on a single spot 27

Fig. S18. Extracting windows of *m/z* and mobility in Fig. 6 28

Fig. S19. MALDI TIMS MSI of endogenous chiral amino acids in a mouse brain tissue section 29

1.1 Animal preparation

C57Bl/6 mice were obtained from Laboratory Animal Service Centre at The Chinese University of Hong Kong (Hong Kong SAR, China). The mice were housed in standard conditions with a 12h light /12h dark cycle and fed with sterilized water and standard laboratory feed. The mice were sacrificed, and brain tissues were dissected and stored at -80 ℃ prior to metabolites extraction and section preparation. Experimental protocols were approved by the Hong Kong Baptist University Committee on the Use of Human and Animal Subjects in Teaching and Research.

1.2 Amino acids extraction from mouse brain tissue

The preparation of mouse brain extracts followed the workflow previously reported[1].50 mg of brain tissue samples were homogenized in 500 μL of ice-cold MeOH/H_2O (4:1, v: v) using Bullet Blender Storm 24 (Next Advance, Troy, NY, USA). 300 μL of homogenate was extracted and mixed with 180 μL of chloroform and 60 μL of H_2O. The mixture was vortexed for 1 min, rested for 5 min to equilibrate to room temperature, and then centrifuged at 20 000 g for 10 min at 4 ℃. The up layer was collected, transferred to a 1.5 mL microcentrifuge tube, and stored at -80 ℃ prior to the FDAA derivatization reaction.

1.3 Sample preparation for MALDI MSI

Mouse brain tissue was cryosectioned at 10 μm using CryoStar Nx70 cryostat (Thermal Fisher Scientific, Walldorf, Germany) at a chamber temperature of -20 °C. The tissue cryosections were then transferred onto an indium tin oxide (ITO)-coated glass slide (2.5 cm x 7.5 cm, Delta Technologies, Loveland, CO, USA). The tissue sections were rinsed with cold chloroform for 15s at -18 °C to remove lipids from the tissue section, which was reported to improve the detection of small molecule metabolites[2]. Adjacent tissue slides were stained with hematoxylin and eosin (H&E staining), and optical images were captured using Confocal FLIM Imaging System (Nikon C2si Plus, Japan).

The mixing solution of 38 AAs (About 212 μM of each AA) was prepared in an aqueous $NaHCO_3$ (100 mM) solution and then was diluted twice using MeOH. Half of the brain section

was covered with a coverslip (Corning, NY, USA) to prevent standards from depositing on this half. The AA mixture was applied to the tissue section covering the dimensions of 2.6 cm x 3 cm using a custom-built matrix deposition system. The setup of the spray system is similar to our previously reported electrospray deposition device[3]. The AA mixture was delivered by a springe pump (Chemyx Fusion 101, Chemyx, Stafford, TX, USA) with a flow rate of 10 μL/min for 30 min through a 100 μm i.d. quartz capillary (IDEX Corp., Northbrook, IL, USA) to the spray nozzle with N_2 sheath gas (99.995 %) of 70 psi heated to 50 °C. The spraying voltage was set to +5 kV using a high voltage power supply (PS 350, Stanford Research Systems, Sunnyvale, CA, USA). The motorized stage was set at a velocity of 1098 mm/min with 1 mm track spacing.

On-tissue FDAA derivatization was performed in a light-proof reaction chamber for 60 min using the homemade deposition system. FDAA (20 mM) in a MeOH/THF (1:1, v/v) solution and an aqueous $NaHCO_3$ (100 mM) solution were delivered at a flow rate of 15 μL/min separately and mixed online in a PEEK tee (IDEX Corp., Northbrook, IL, USA). The sheath gas was set to 35 psi, and the target plate was heated to 45 °C. The deposition dimensions were set to 2.6 cm x 3 cm to ensure at least 0.5 cm spacing between the deposition edge and the tissue on each slide.

After derivatization, a DHAP solution (5 mg/mL) in MeOH was applied onto the tissue section using the same settings as the deposition of AAs, except that the flow rate, thickness and dimensions were changed to 30 μL/min, 13 cycles and 2.6 cm x 3 cm, respectively.

Table S1. Parameters of direct-infusion TIMS-MS in Tune

Transfer			
Funnel 1 RF	350.0 V_{pp}	isCID Energy	0.0 eV
Funnel 2 RF	350.0 V_{pp}	Deflection Delta	70.0 V
Multipole RF	350.0 V_{pp}		
Quadrupole			
Ion Energy	5.0 eV	Low Mass	300.00 *m/z*
Collision Cell			
Collision Energy	10.0 eV	Collision RF	1800.0 V_{pp}
Focus Pre TOF			
Transfer Time	60.0 μs	Pre Pulse Storage	8.0 μs

Table S2. Parameters of MALDI TIMS-MS in Tune

Transfer			
MALDI Plate Offset	50.0 V	Deflection 1 Delta	70.0 V
Funnel 1 RF	200.0 V_{pp}	isCID Energy	0.0 eV
Funnel 2 RF	220.0 V_{pp}	Multipole RF	220.0 V_{pp}
Quadrupole			
Ion Energy	5.0 eV	Low Mass	150.00 *m/z*
Collision Cell			
Collision Energy	10.0 eV	Collision RF	1500.0 V_{pp}
Focus Pre TOF			
Transfer Time	60.0 μs	Pre Pulse Storage	5.0 μs

Table S3. Parameters in Tune for Q-TOF only mode (TIMS was switched off)

Transfer			
Funnel 1 RF	350.0 V_{pp}	isCID Energy	0.0 eV
Funnel 2 RF	350.0 V_{pp}	Deflection Delta	70.0 V
Multipole RF	350.0 V_{pp}		
Quadrupole			
Ion Energy	5.0 eV	Low Mass	50.00 *m/z*
Collision Cell			
Collision Energy	10.0 eV	Collision RF	400.0 V_{pp}
Focus Pre TOF			
Transfer Time	60.0 μs	Pre Pulse Storage	8.0 μs

Table S4. Elution orders and R of FDAA-AA diastereomers obtained from TIMS-MS

FDAA-AA	First eluting AA	R_D	R_L
Ala	L	123.3	147.5
Arg	L	146.7	134.4
Asn	L	112.7	148.1
Asp	L	93.8	168.4
Cys[a]	L	154.7	115.7
Cys[b]	D	184.9	114.4
Gln	L	165.4	164.9
Glu	L	122.4	146.9
His	L	176.5	172.0
Leu	L	144.3	164.6
Ile	L	146.5	164.6
Lys[a]	L	150.3	153.8
Lys[b]	L	162.0	161.4
Met	L	140.9	147.4
Phe	L	157.9	134.0
Pro	L	140.0	181.4
Ser	L	137.4	141.5
Thr	L	157.8	160.8
Trp	L	154.0	163.5
Tyr	L	163.5	107.1
Val	L	172.7	132.4

[a]mono-derivatives

[b]bis-derivatives

Table S5. Lists of product ions from precursor ions $[FDAA\text{-}AA + 2Na - H]^+$ corresponding to Fig. S16.

Amino acids	precursor ions	Product ions (excluding 'R[1]' group)	Product ions (including 'R' group)
Ala	386.07	87.98, 91.97, 196.01, 208.01, 265.03, 294.02, 309.04	324.07, 342.08
Ser	402.06	87.98, 91.97, 196.01,208.01, 265.03, 310.05	340.06, 358.07
Pro	412.08	87.98, 91.97, 196.01, 208.01	350.09
Val	414.10	87.98, 91.97, 196.01, 208.01, 265.03, 294.02, 309.04	352.10, 370.11
Thr	416.08	87.98, 91.97, 196.01, 208.01, 265.03, 294.02, 310.05	
Ile, Leu	428.12	87.98, 91.97, 196.01, 208.01, 265.03, 294.02, 309.04	366.11, 384.12
Asn	429.07	87.98, 91.97, 196.01, 265.03, 294.02, 309.04, 310.05	367.07
Gln	443.09	87.98, 91.97, 196.01, 208.01, 294.02, 309.04, 310.05	381.09
Met	446.07	87.98, 91.97, 196.01	384.07
His	452.09	87.98, 91.97, 196.01, 208.01, 265.03, 294.02, 309.04, 310.05	390.09
Phe	462.10	87.98, 91.97, 196.01, 208.01, 265.03, 294.02, 309.04, 310.05	400.10
Arg	471.13	87.98, 91.97, 196.01, 208.01, 265.03, 294.02, 309.04	409.13
Tyr	478.09	87.98, 91.97, 196.01, 208.01, 265.03, 294.02, 309.05, 310.05	416.10
Trp	501.11	87.98, 91.97, 196.01, 208.01, 265.03, 294.02, 309.04, 310.05	

[1]a side chain specific to each AA

Table S6. Extracting windows of *m/z* and mobility in Fig. S18

FDAA-AA	*m/z* up limit	*m/z* lower limit	$1/k_0$ up limit	$1/k_0$ lower limit
D-Ala	386.059	386.079	0.8960	0.9080
L-Ala	386.059	386.079	0.9120	0.9260
D-Arg	471.122	471.142	0.9895	0.9975
L-Arg	471.122	471.142	0.9985	1.0065
D-Asn	429.064	429.084	0.9265	0.9365
L-Asn	429.064	429.084	0.9380	0.9480
D-Asp	430.048	430.068	0.9200	0.9360
L-Asp	430.048	430.068	0.9400	0.9520
D-Gln	443.079	443.099	0.9460	0.9560
L-Gln	443.079	443.099	0.9580	0.9680
D-Glu	444.063	444.083	0.9500	0.9580
L-Glu	444.063	444.083	0.9610	0.9670
D-His	452.080	452.100	0.9580	0.9660
L-His	452.080	452.100	0.9810	0.9880
D-Leu	428.105	428.125	0.9700	0.9706
L-Leu	428.105	428.125	0.9830	0.9890
D-Ile	428.105	428.125	0.9580	0.9680
L-Ile	428.105	428.125	0.9770	0.9810
D-Lys (di)	695.164	695.184	1.1275	1.1415
L-Lys (di)	695.164	695.184	1.1500	1.1640
D-Met	446.062	446.082	0.9450	0.9590
L-Met	446.062	446.082	0.9650	0.9790
D-Phe	462.089	462.109	0.9620	0.9740
L-Phe	462.089	462.109	0.9760	0.9900
D-Pro	412.074	412.094	0.9140	0.9240
L-Pro	412.074	412.094	0.9320	0.9440
D-Ser	402.055	402.075	0.8980	0.9100
L-Ser	402.055	402.075	0.9120	0.9240
D-Thr	416.069	416.089	0.9220	0.9320
L-Thr	416.069	416.089	0.9320	0.9440
D-Trp	501.100	501.120	1.0060	1.0200
L-Trp	501.100	501.120	1.0200	1.0340
D-Tyr[1]	478.084	478.104	0.9860	0.9980
L-Tyr	478.084	478.104	1.0000	1.0140
D-Tyr[2]	478.084	478.104	1.0280	1.0420
D-Val	414.090	414.110	0.9350	0.9450
L-Val	414.090	414.110	0.9500	0.9620

di: bis-derivatives

[1]first eluted mobility peak of D-Tyr

[2]second eluted mobility peak of D-Tyr

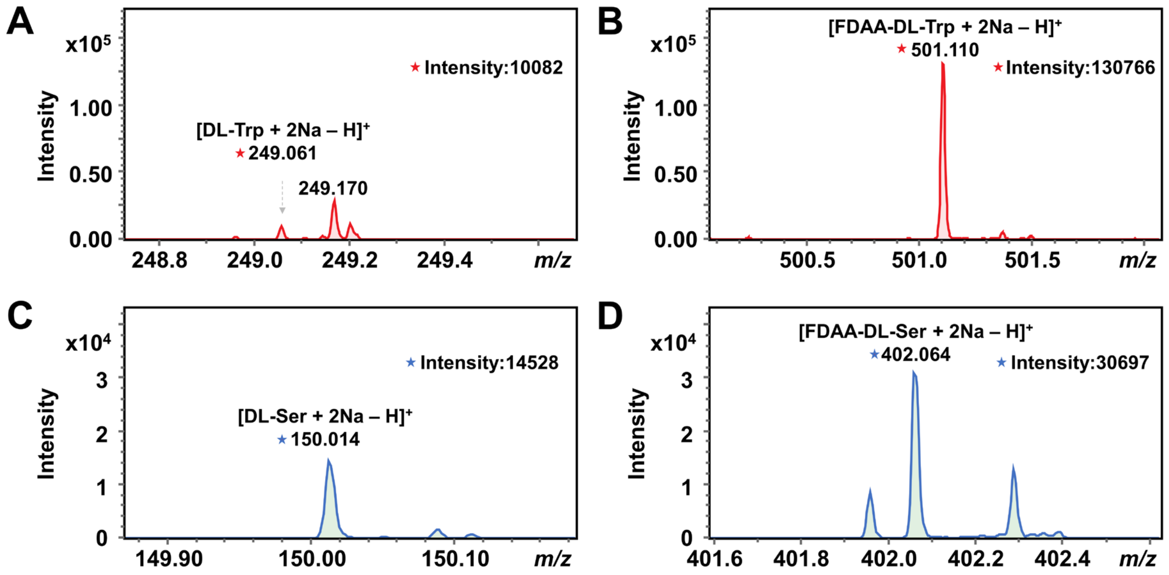

Fig. S1. Mass spectra of DL-Trp and DL-Ser (D: L = 1: 1) after FDAA derivatization for the comparison of relative ion intensity (RI) between product and reactant ions. (A) unlabeled Trp. (B) FDAA-labeled Trp. (C) unlabeled Ser. (D) FDAA-labeled Ser. Subfigures with the same color were obtained from the same mass spectrum. In order to observe ions at low *m/z*, TIMS was switched off and parameters in Tune was optimized in Table S3.

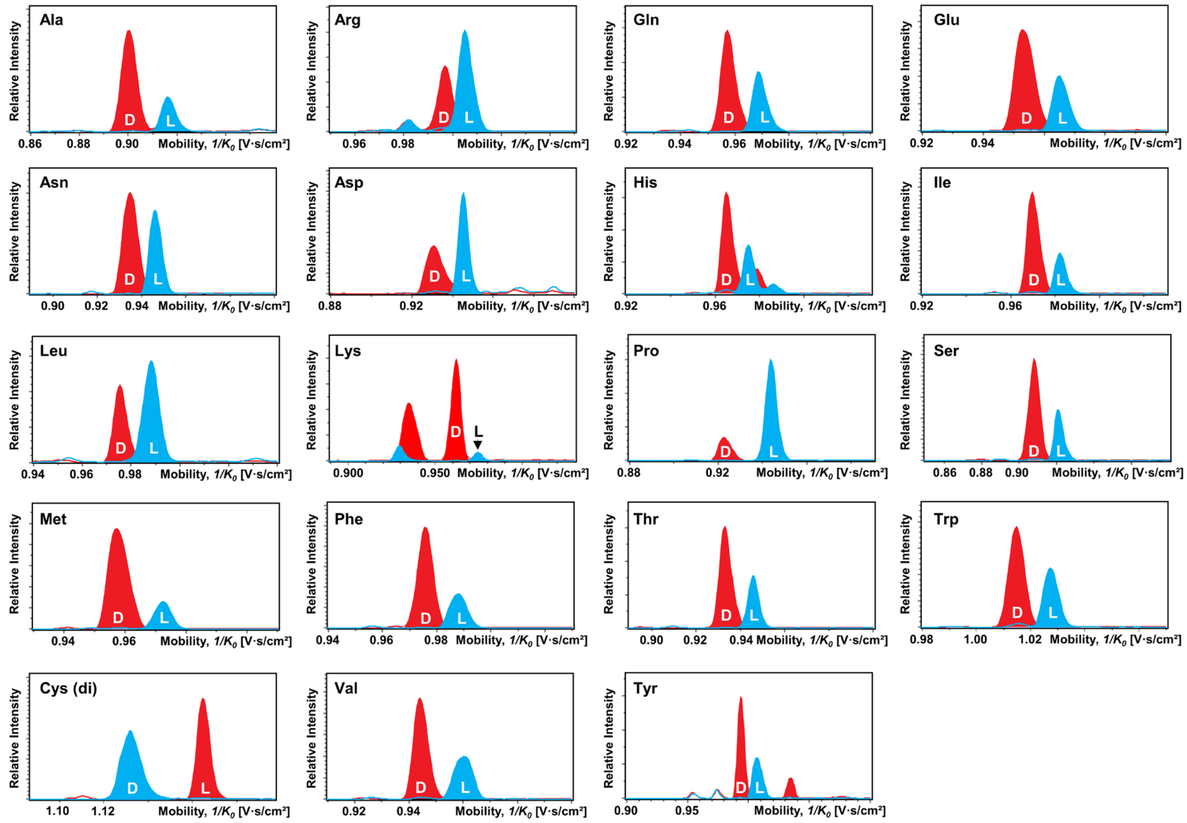

Fig. S2. Overlay EIMs of a single chiral amino acid standard after FDAA derivatization. The corresponding D-forms and L-forms were measured by TIMS-MS separately.

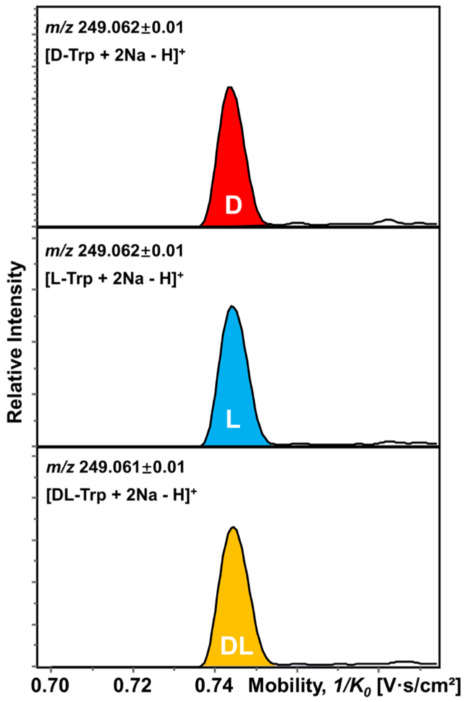

Fig. S3. EIMs of Trp standards (100 μM) before derivatization. D form, L form, and racemic mixture were measured separately.

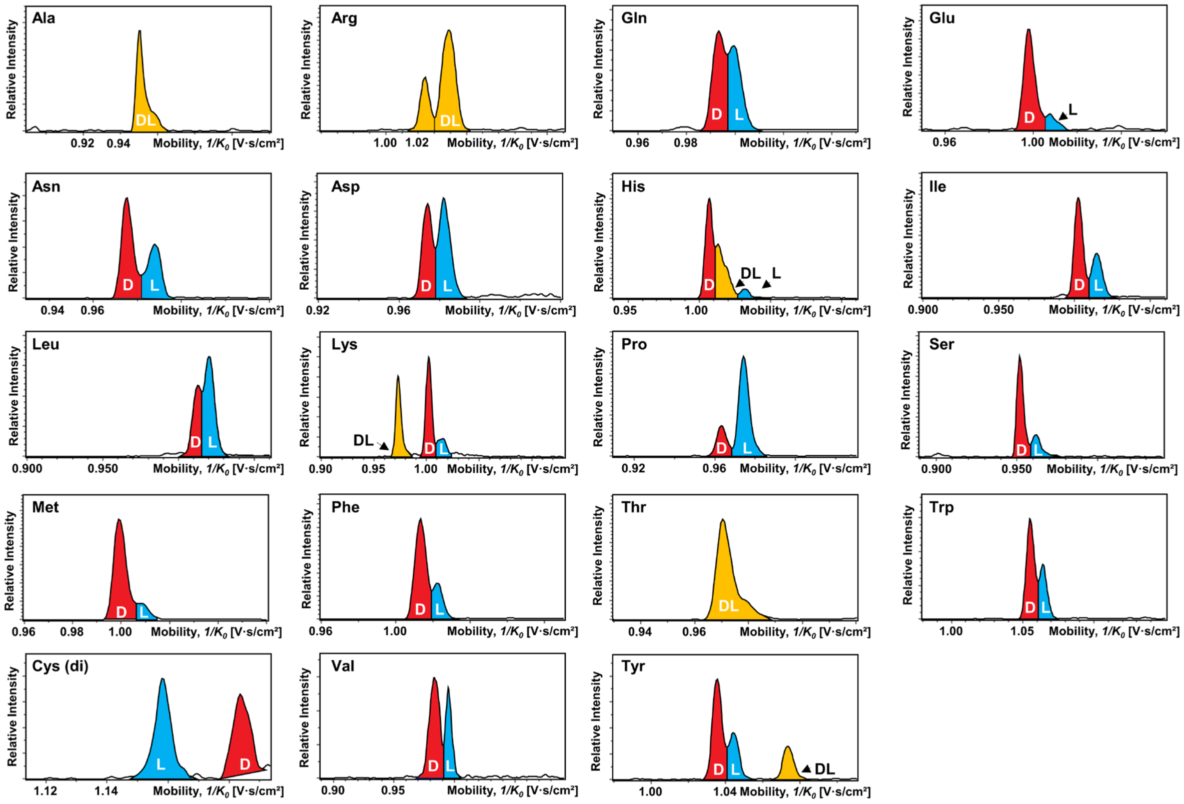

Fig. S4. EIMs of 19 pairs of proteinogenic AA (D:L = 1:1) standards after FDVA derivatization.

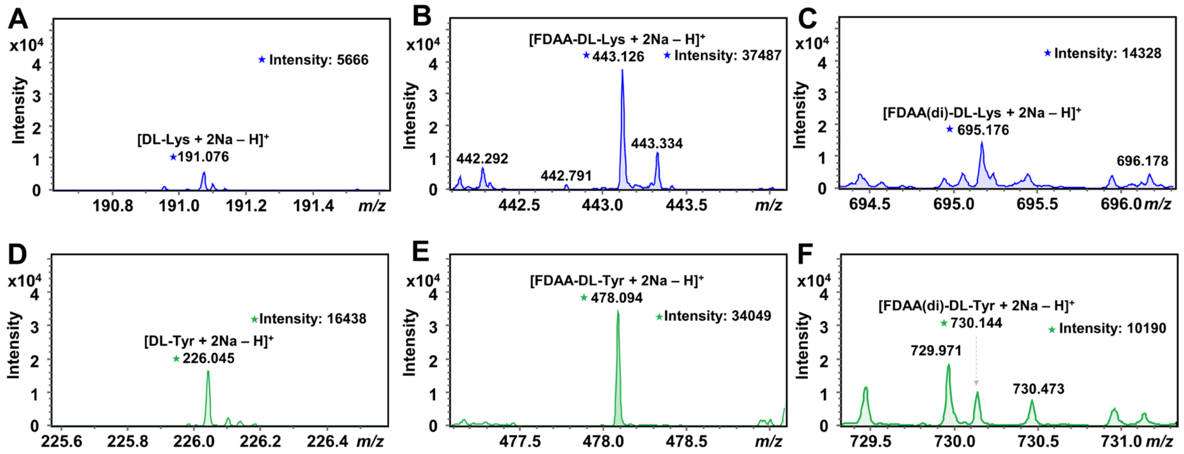

Fig. S5. Mass spectra of AAs with multiple reaction sites (DL-Lys and DL-Tyr) obtained after FDAA derivatization for the comparison of relative ion intensity (RI) between mono-derivative and bis-derivative ions. (A) unlabeled Lys. (B) FDAA-labeled Lys. (C) Bis-derivatives of Lys. (D) unlabeled Tyr. (E) FDAA-labeled Tyr. (F) Bis-derivatives of Tyr. Subfigures with the same color were obtained from the same mass spectrum. In order to observe ions at low *m/z*, TIMS was switched off and parameters in Tune was optimized in Table S3.

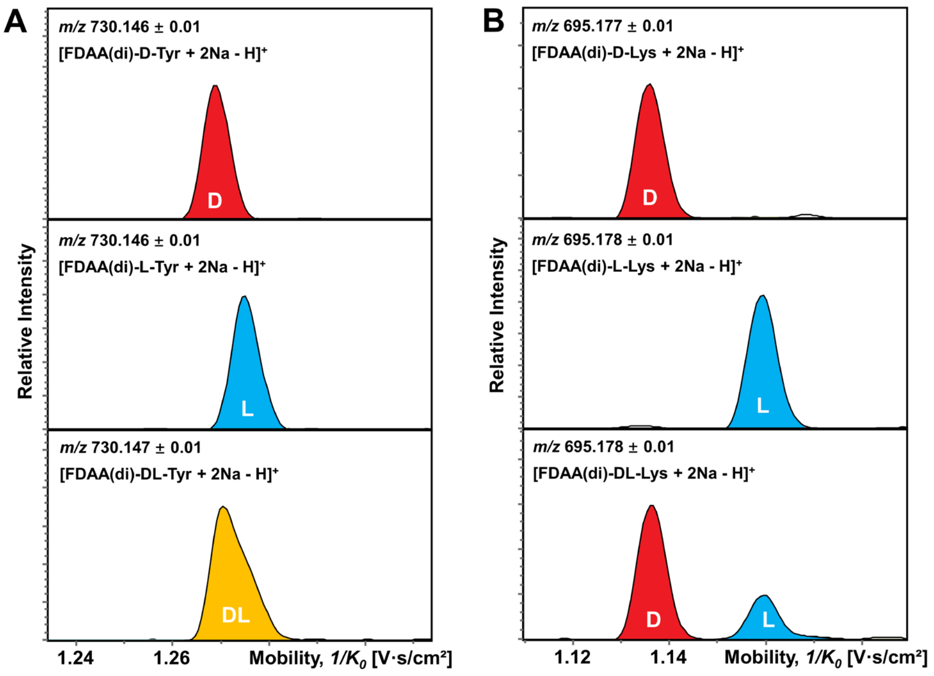

Fig. S6. EIMs of bis-derivatives of (A) Tyr and (B) Lys.

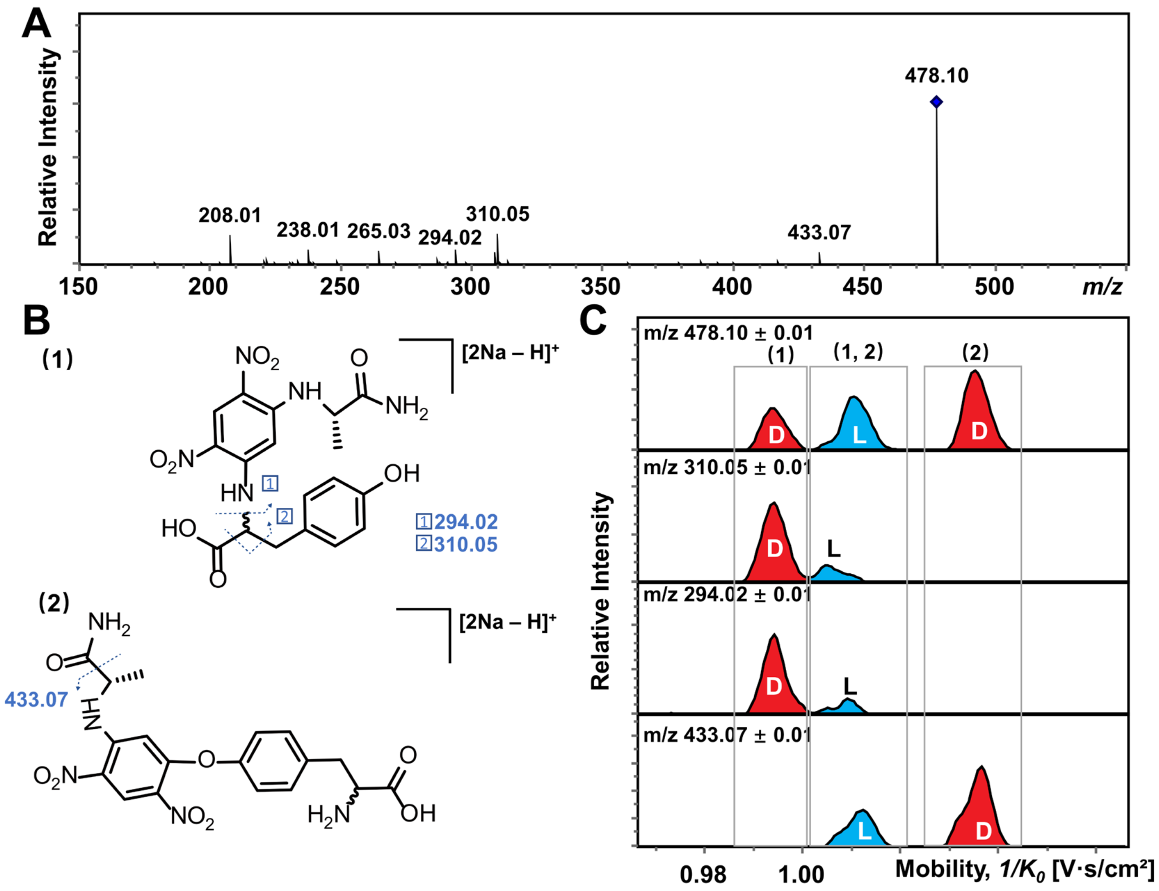

Fig. S7. Determination of the reaction site of Tyr with FDAA by the combination of TIMS and tandem mass spectrometry (MS/MS). (A) MS/MS spectrum of FDAA-DL-Tyr. (B) Putative fragmentation sites of Tyr after the reaction of FDAA with (1) α-amino group and (2) hydroxyl group. (C) EIMs of product ions obtained from TIMS-MS/MS of selected ions at *m/z* 478.10. Product ions showing the same mobility are derived from the same parent ions, and therefore, product ions at *m/z* 310. 05 and 294.02 are produced from structure (1) and product ions at *m/z* 433.07 are produced from structure (2).

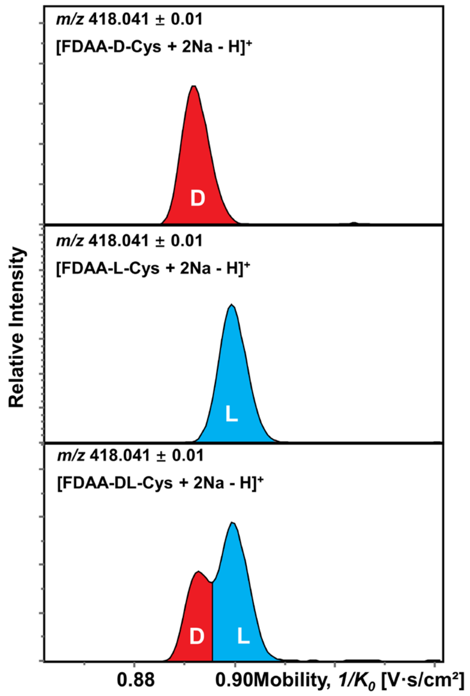

Fig. S8. EIMs of mono-derivatives of Cys.

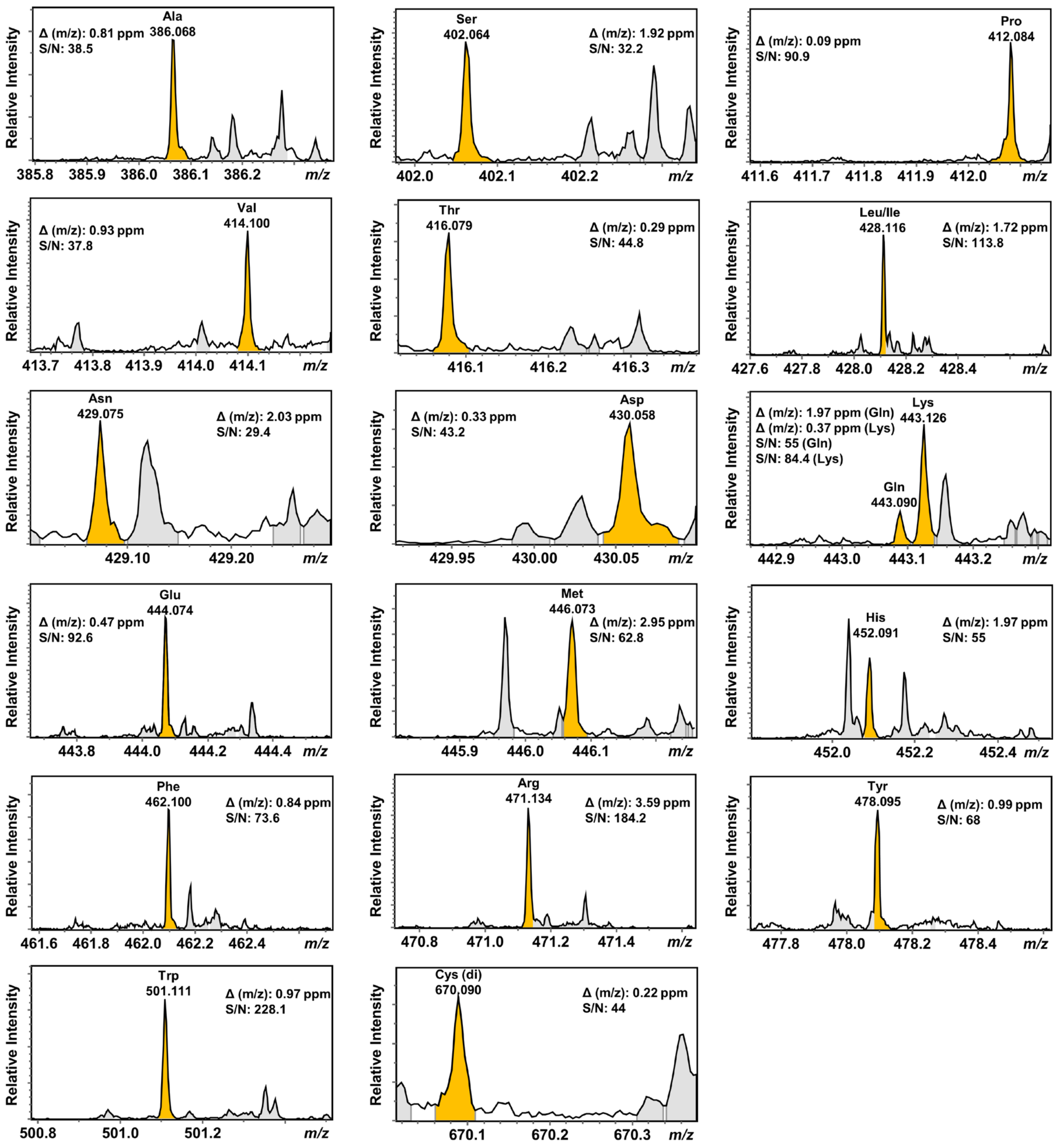

Fig. S9. Mass spectra of 38 mixed amino acids corresponding to Fig. 4. The mass deviation and signal to noise ratio (S/N) of highlighted peaks are presented as well.

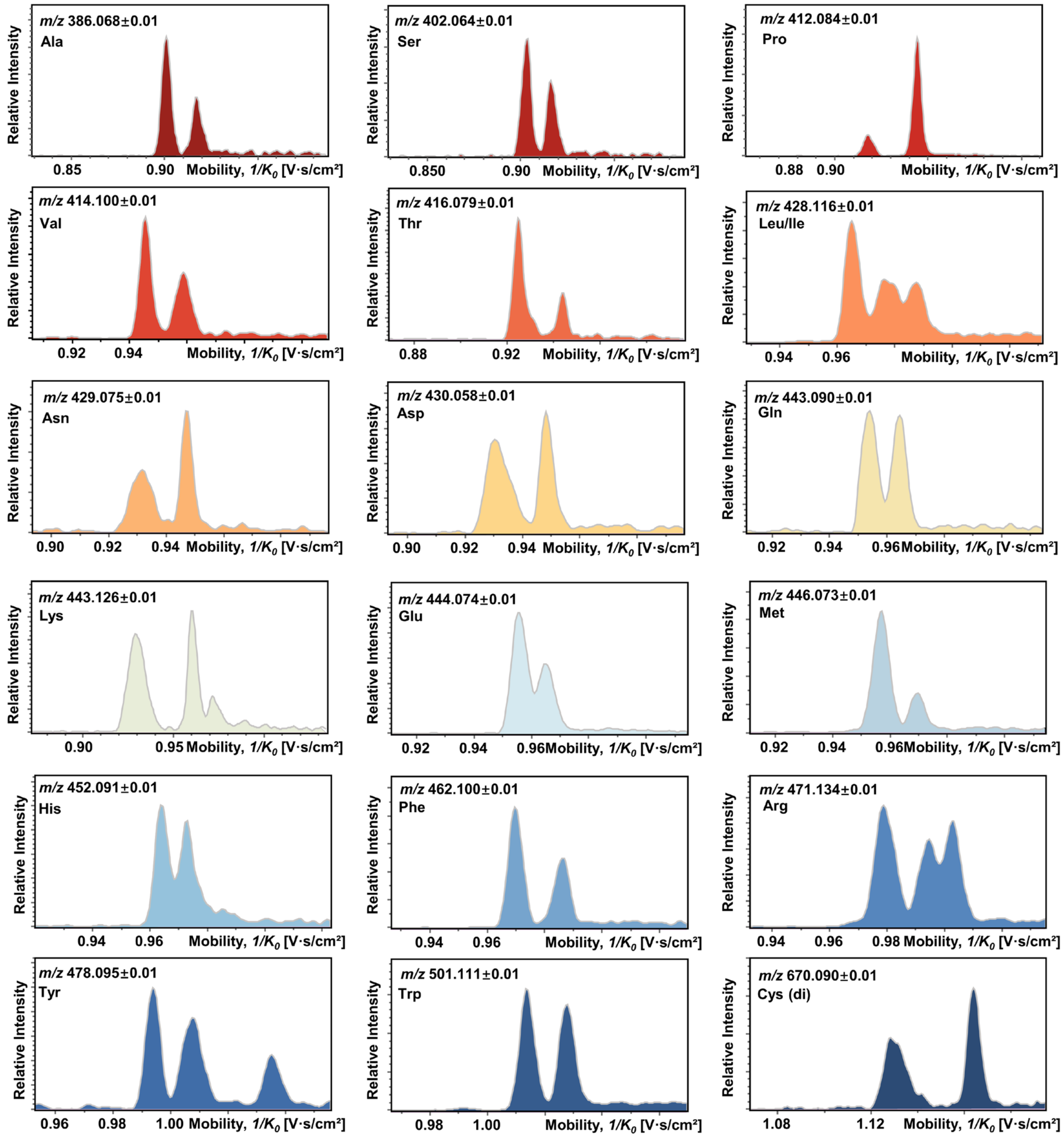

Fig. S10. Separately listed EIMs of the mixture of 38 chiral AAs corresponding to Fig. 4.

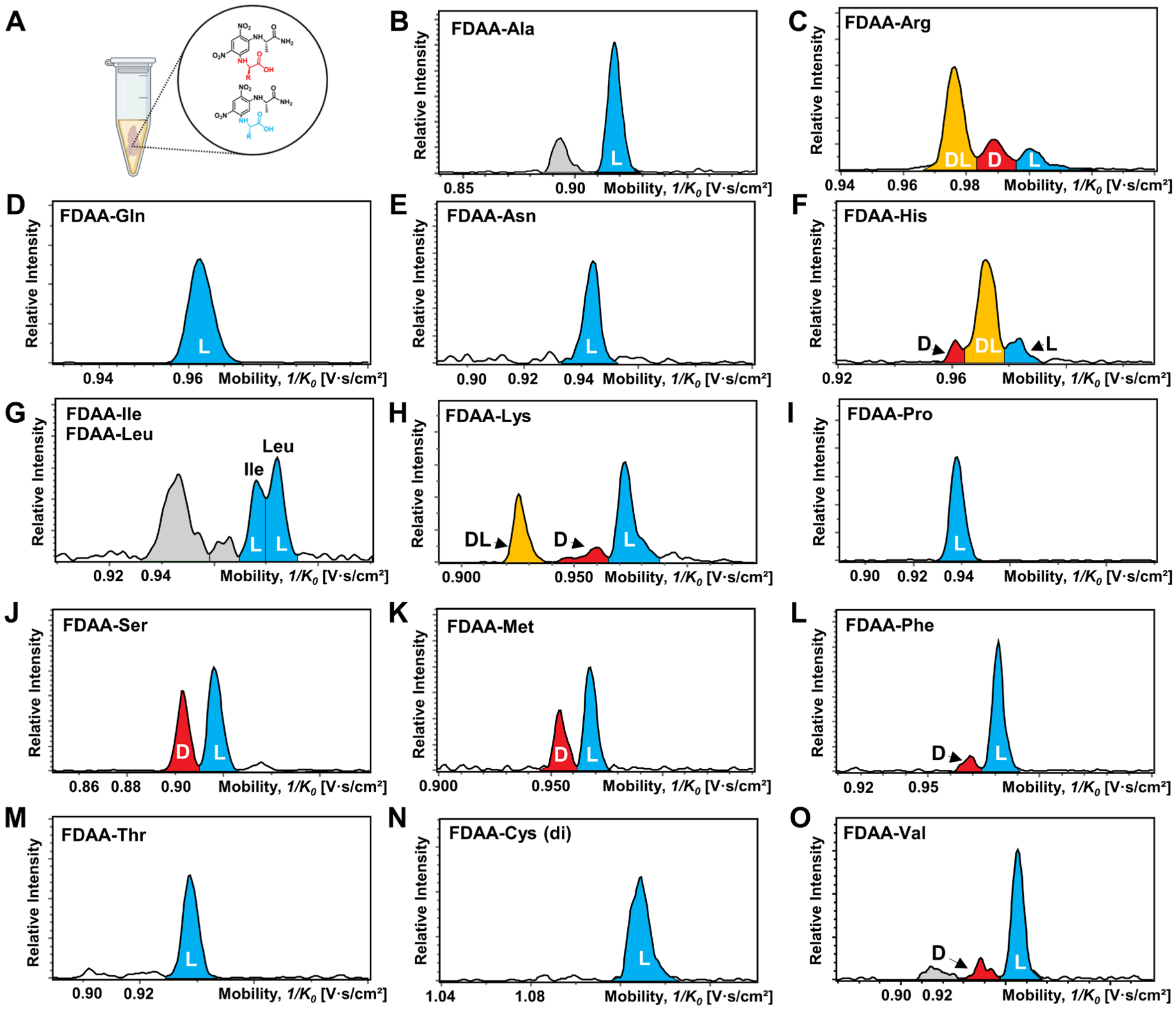

Fig. S11. EIMs of 15 AAs measured in the mouse brain extracts after FDAA derivatization. (A) Schematic diagram of mouse brain extracts, which was created with BioRender.com. (B-O) EIMs of FDAA-AA.

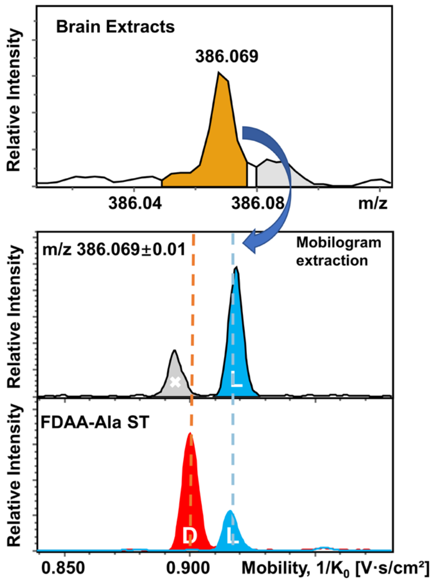

Fig. S12. An example of amino acid (Ala) identification in mouse brain extracts using FDAA derivatization by TIMS-MS. Mobility distribution in the EIM of *m/z* 386.069 ± 0.01 is compared with FDAA-Ala pure standard. Peaks in red, blue, and grey traces represent D-AA, L-AA, and interference ions, respectively.

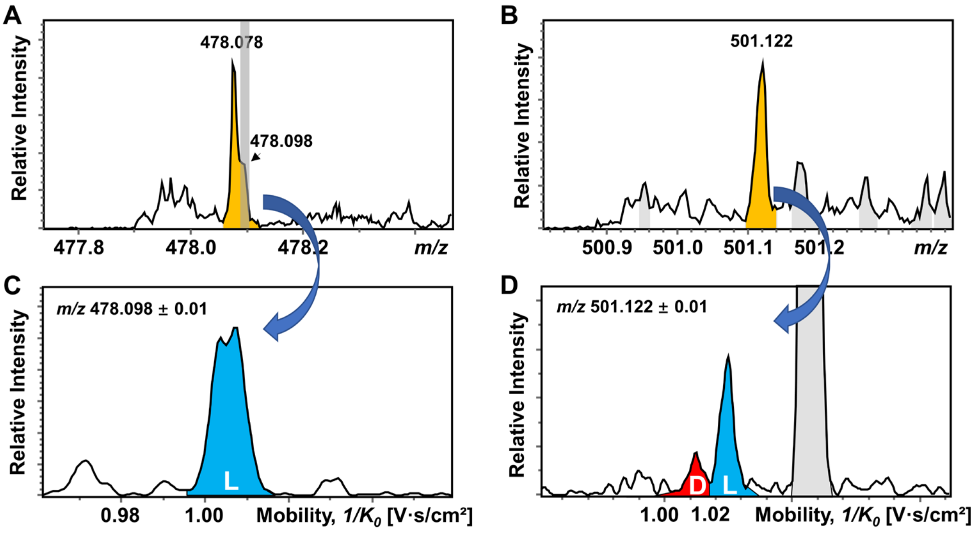

Fig. S13. Mass spectra and EIMs of Tyr and Trp measured in the mouse brain extracts after FDAA derivatization. (A) Mass spectrum corresponding to FDAA-Tyr. (B) Mass spectrum corresponding to FDAA-Trp. (C) EIMs of *m/z* 478.098 ± 0.01. (D) EIMs of *m/z* 501.122 ± 0.01. Peaks in red, blue, and grey traces represent D-AA, L-AA, and interference ions, respectively. While the *m/z* of target analytes are overlapped with an interference peak, the target analytes can be recognized in the EIMs.

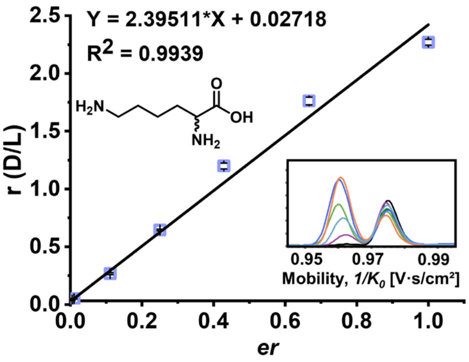

Fig. S14. The plot of peak area ratio against enantiomeric ratio (D/L) of Lys after single FDAA derivatization. The corresponding extracted ion mobilograms of D/L-Lys at different mixing ratios are embedded. Four technical replications of the TIMS-MS analysis were performed.

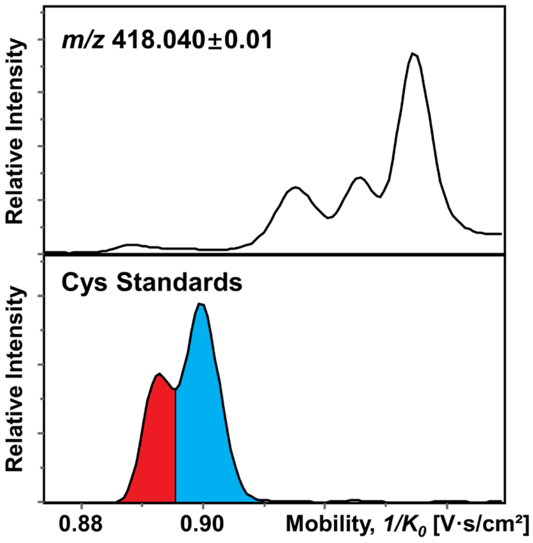

Fig. S15. EIMs of *m/z* 418.040 ± 0.01 measured in the mouse brain section and Cys standards after FDAA derivatization.

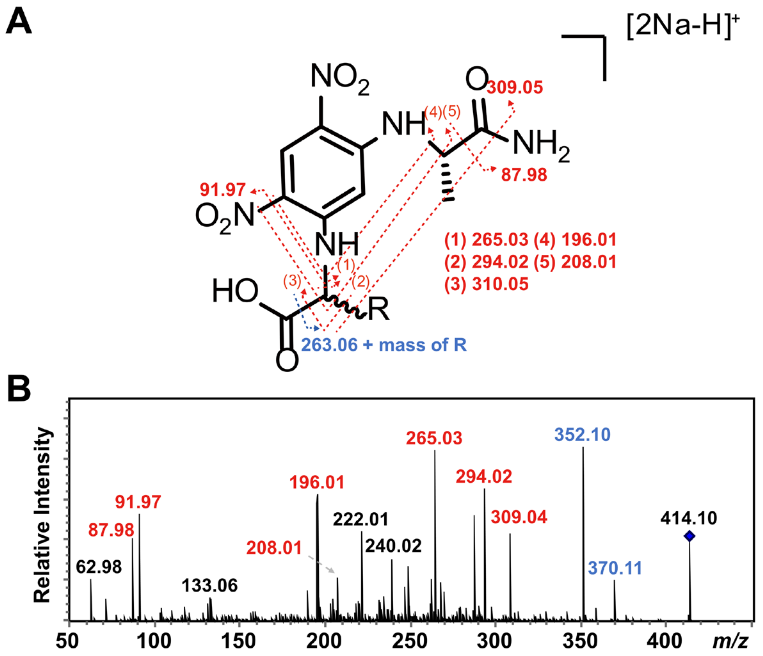

Fig. S16. Confirmation of FDAA-AAs observed in Fig. 6 by tandem mass spectrometry (MS/MS). (A) Putative fragmentation sites of ions [FDAA-AA + 2Na – H]$^+$, where 'R' represents a side chain specific to each AA. (B) A representative MS/MS spectrum of ions [FDAA-Val + 2Na – H]$^+$.

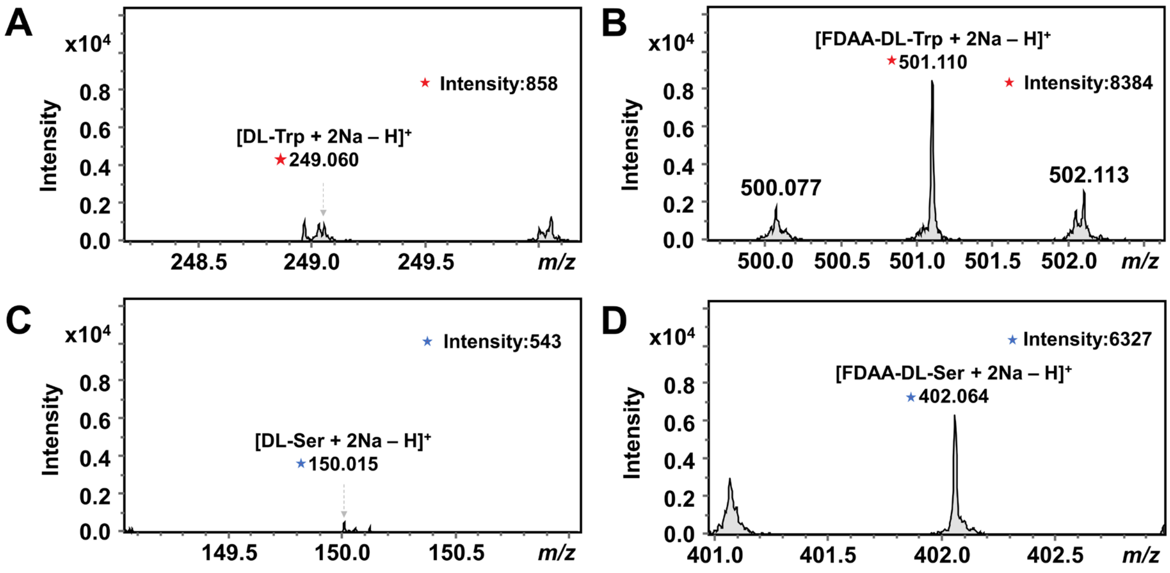

Fig. S17. Mass spectra of DL-Trp and DL-Ser after FDAA derivatization acquired by MALDI on a single spot for the comparison of relative ion intensity (RI) between product and reactant ions. (A) unlabeled Trp. (B) FDAA-labeled Trp. (C) unlabeled Ser. (D) FDAA-labeled Ser. All subfigures were obtained from the same mass spectrum. In order to observe ions at low *m/z*, TIMS was switched off and parameters in Tune was optimized in Table S3.

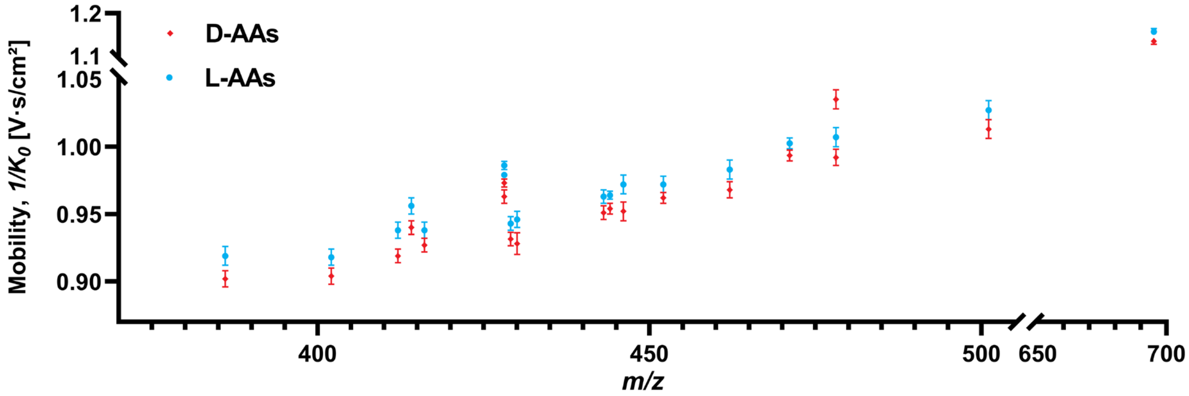

Fig. S18. Extracting windows of *m/z* and mobility in Fig. 6. Detailed values are listed in Table S6. The windows are set based on that no mutual interference occurs for ion images.

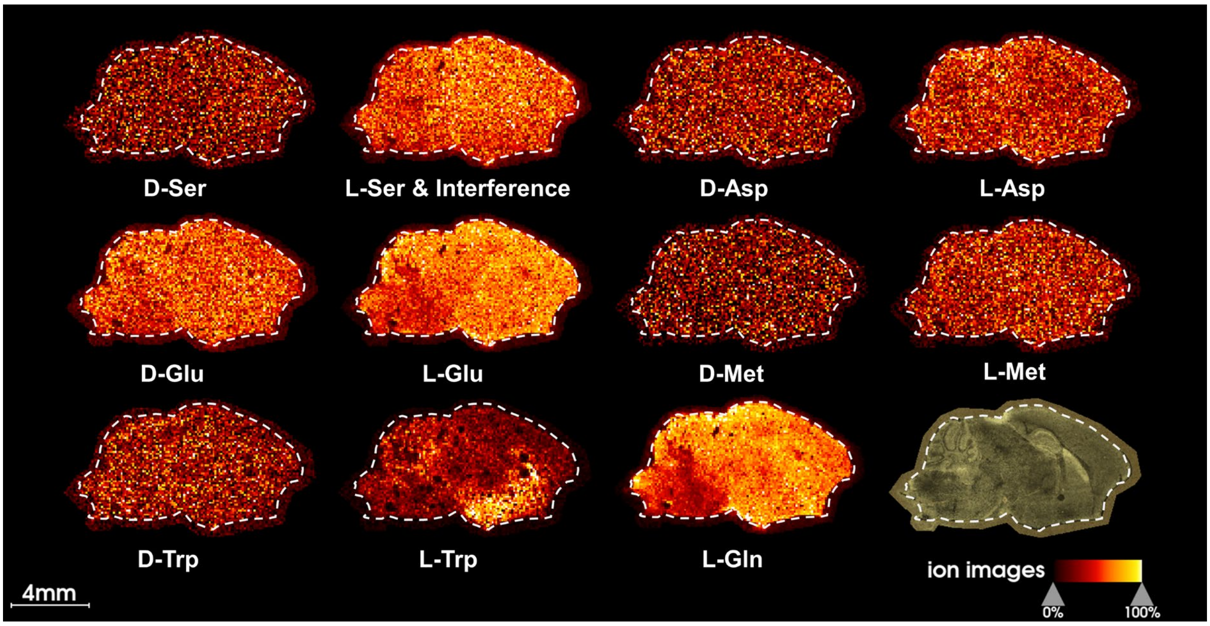

Fig. S19. MALDI TIMS MSI of endogenous chiral amino acids in a mouse brain tissue section.

References

1 C. Zhao, P. Xie, H. Wang and Z. Cai, *J. Hazard. Mater.*, 2018, **358**, 503–507.
2 H. Yang, W. Ji, M. Guan, S. Li, Y. Zhang, Z. Zhao and L. Mao, *Metabolomics*, 2018, **14**, 50.
3 J. Wang, S. Qiu, S. Chen, C. Xiong, H. Liu, J. Wang, N. Zhang, J. Hou, Q. He and Z. Nie, *Anal Chem*, 2015, **87**, 422-430.
